# Promising results with image guided intensity modulated radiotherapy for muscle invasive bladder cancer

**DOI:** 10.1186/s13014-015-0499-0

**Published:** 2015-09-25

**Authors:** D. Whalley, H. Caine, P. McCloud, L. Guo, A. Kneebone, T. Eade

**Affiliations:** Northern Sydney Cancer Centre, Radiation Oncology, Royal North Shore Hospital, Reserve Road, St Leonards, Sydney, NSW 2065 Australia; McCloud Consulting Group, 7-9 Merriwa Street, Gordon, NSW 2072 Australia; Northern Clinical School, University of Sydney, Camperdown, NSW 2050 Australia

## Abstract

**Aim:**

To describe the feasibility of image guided intensity modulated radiotherapy (IG-IMRT) using daily soft tissue matching in the treatment of bladder cancer.

**Methods:**

Twenty-eight patients with muscle-invasive carcinoma of the bladder were recruited to a protocol of definitive radiation using IMRT with accelerated hypofractionation with simultaneous integrated boost (SIB). Isotropic margins of .5 and 1 cm were used to generate the high risk and intermediate risk planning target volumes respectively. Cone beam CT (CBCT) was acquired daily and a soft tissue match was performed. Cystoscopy was scheduled 6 weeks post treatment.

**Results:**

The median age was 83 years (range 58-92). Twenty patients had stage II or III disease, and eight were stage IV. Gross disease received 66 Gy in 30 fractions in 11 patients (ten with concurrent chemotherapy) or 55 Gy in 20 fractions for those of poorer performance status or with palliative intent. All patients completed radiation treatment as planned. Three patients ceased chemotherapy early due to toxicity. Six patients (21 %) had acute Grade ≥ 2 genitourinary (GU) toxicity and six (21 %) had acute Grade ≥ 2 gastrointestinal (GI) toxicity. Five patients (18 %) developed Grade ≥2 late GU toxicity and no ≥2 late GI toxicity was observed.

Nineteen patients underwent cystoscopy following radiation, with complete response (CR) in 16 cases (86 %), including all patients treated with chemoradiotherapy. Eight patients relapsed, four of which were local relapses. Of the patients with local recurrence, one underwent salvage cystectomy. For patients treated with definitive intent, freedom from locoregional recurrence (FFLR) and overall survival (OS) was 90 %/100 % for chemoradiotherapy versus 86 %/69 % for radiotherapy alone.

**Conclusion:**

IG- IMRT using daily soft tissue matching is a feasible in the treatment of bladder cancer, enabling the delivery of accelerated synchronous integrated boost with good early local control outcomes and low toxicity.

## Introduction

Muscle Invasive bladder cancer presents a unique set of treatment related challenges in what is typically an older patient group with significant medical co-morbidity. Radical surgery with cystectomy and pelvic lymph node dissection poses many challenges in this group of patients with high perioperative risks and potential ongoing morbidity in relation to sexual dysfunction and the physical and psychological impact of urinary diversion [1–3]. Bladder preservation, in which radiation is delivered with concurrent chemotherapy following maximal trans-urethral resection of bladder tumour (TURBT), is an alternative to surgical management, with similar 5 year overall survival rates of 48-65 % accounting for stage and co-morbidity [4–6]. However it remains underutilised, with patterns of care studies showing that aggressive radiation-based treatment is offered at a considerably lower rate compared with cystectomy [7, 8].

One of the barriers to greater utilisation of radiotherapy is perhaps the technical challenges with delivering this treatment. The distortable nature of the bladder, variable bladder filling, as well as motion of other pelvic organs all contribute to uncertainties with respect to target coverage [9–11]. The use of image guidance and adaptive therapy is potentially of great value in this setting by allowing the reduction of planning target volume (PTV) margins and improved target coverage [10, 12–14]. Another challenge lies in the radiobiologic characteristics of bladder cancer. There is a narrow therapeutic window with respect to irradiation of the bladder due to the risk of radiation toxicity to the bladder itself, as well as adjacent normal tissues [15, 16]. A radiation dose response has been established in the treatment of bladder cancer, but the wide margins required for standard conformal techniques are dose limiting [17, 18]. Furthermore, there is some evidence that urothelial carcinoma displays sensitivity to overall treatment time, with accelerated repopulation occurring at 5-6 weeks after commencing radiation [19].

IMRT is of considerable interest in this regard due to the superior conformality and normal tissue sparing demonstrated with its use in other pelvic malignancies, most notably prostate and cervical cancer [20, 21]. Its application in bladder cancer is less frequently described, but is appealing due to the potential to dose escalate gross disease using an integrated boost technique, and shorten overall treatment time. The concern with IMRT is of geographic miss; however with daily on treatment soft tissue alignment with CBCT it is possible to overcome this obstacle. In 2010, the Northern Sydney Cancer Centre developed a protocol of IG-IMRT with daily soft tissue matching for the treatment of patients with bladder cancer. Here, we report our experience with this technique in a cohort of 28 patients treated from 2010-2013.

## Methods

### Patient selection

Approval for this study was obtained from the Northern Sydney Local Health District Human Research Ethics Committee. Potential candidates for the IG-IMRT protocol were identified at the genitourinary cancer multidisciplinary meeting attended by members of the departments of Urology, Pathology, Radiology, Medical Oncology and Radiation Oncology. Patients were eligible for treatment if they had biopsy proven muscle-invasive urothelial or squamous carcinoma of the bladder and were not for definitive cystectomy (either due to inoperable disease or patient preference). Exclusion criteria included previous pelvic radiation, poor performance status (ECOG ≥3) and extensive visceral metastases. Patients with low volume metastases were considered if their life expectancy was estimated to be >6 months.

In addition to diagnostic cystoscopy and biopsy, patients underwent staging with whole body bone scan and CT of the chest, abdomen and pelvis. Unless contraindicated, pelvic multiparametric MRI was performed to aid tumour delineation. In patients being considered for curative chemoradiation (CRT), maximal TURBT was performed ≤6 weeks prior to commencing radiation treatment. When possible, patients also had 3-4 gold markers inserted near the tumour bed under cystoscopic guidance. This procedure was performed by their Urologist under general anaesthesia.

### Planning and treatment

After providing written consent for radiation treatment, patients underwent CT simulation. Two image sets were acquired for each patient: one with the patient’s bladder comfortably full, and the second with an empty bladder. Planning CT images were fused with the diagnostic contrast-enhanced CT and MRI to aid tumour volume delineation. As the bladder volume of the MRI commonly differed from the planning CT scan, the MRI was interpreted with the aid of a radiologist to define the anatomical area of the bladder at highest risk of gross residual disease (GTV), which was then contoured. The intermediate risk clinical target volume (IRCTV) was defined as the whole bladder and expanded by 10 mm to the intermediate risk PTV (IRPTV) and the GTV was expanded by 0.5 cm to create the high risk PTV (HRPTV). If treated, the uninvolved obturator, internal and external iliac nodes were contoured as a separate low risk CTV (LRCTV) and then expanded by a 1 cm margin to a low risk PTV (LRPTV).

Most patients were subsequently treated with an empty bladder, but the “full bladder” image set was used in patients when appropriate to separate the small bowel from the gross disease. This decision was made by the treating oncologist before the planning was performed. Radiation was planned and delivered using a 7-10 field sliding-window IMRT technique on a Varian Trilogy (Varian Medical Systems, Palo Alto, CA, USA). Patients were treated with and accelerated moderate hypofractionated schedule with either 20 or 30 fractions, incorporating a simultaneous integrated boost to the GTV if identified. The 30 fraction group included patients with localised disease who were regarded fit enough to receive combined chemo-irradiation. They received 66 Gy, 60 Gy and 54 Gy to the HRPTV, IRPTV and the pelvic nodes respectively. In patients treated with 20 fractions, the doses were 55 Gy, 50 Gy and 45 Gy without concurrent chemotherapy.

Platinum chemotherapy was administered concurrently in suitable candidates. Chemotherapy was cisplatin 35 mg/m^2^ once weekly unless contraindicated when weekly AUC2 carboplatin was used.

**Fig. 1:**
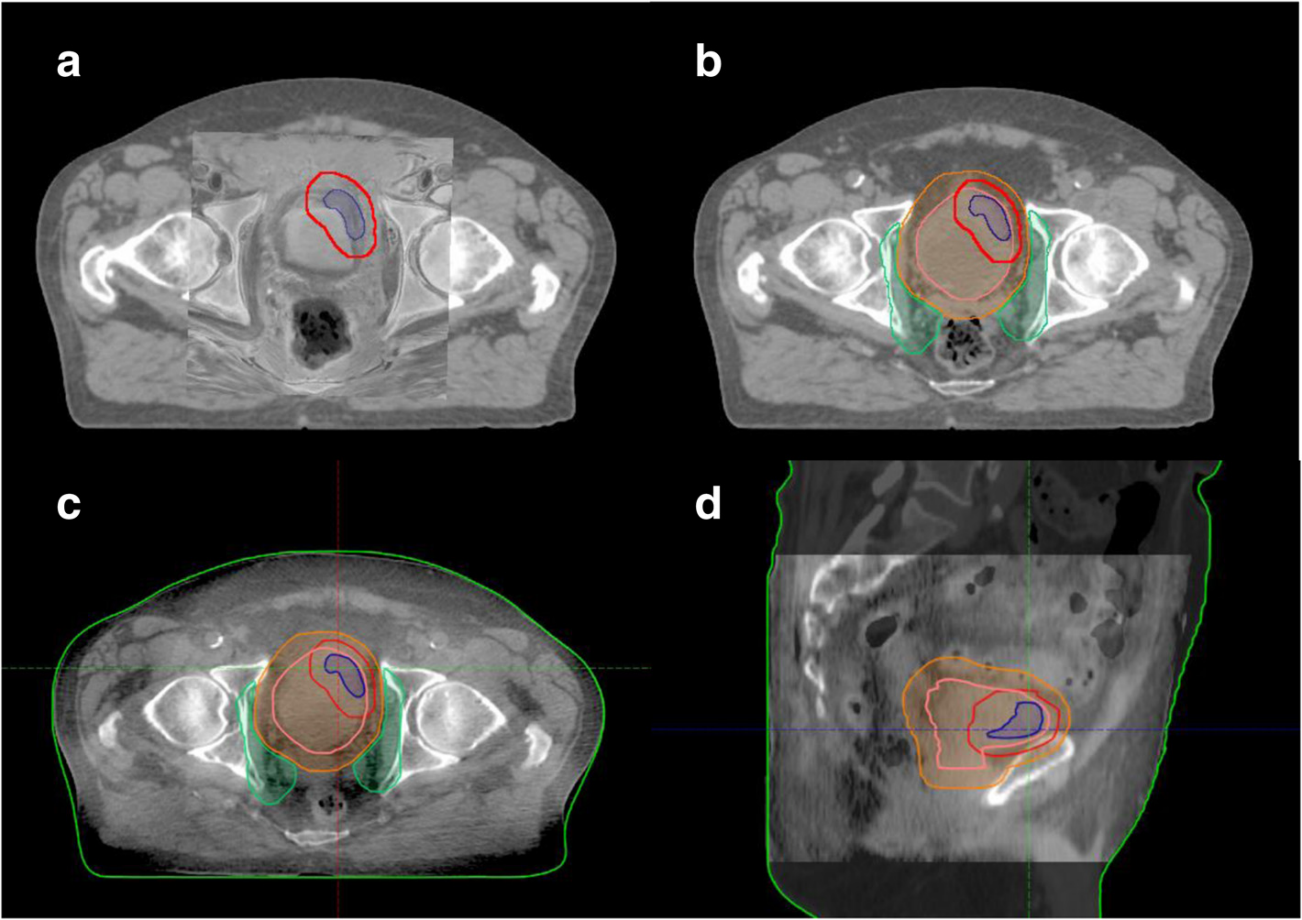
GTV delineation and soft tissue match to GTV on CBCT. Figs. **a** and **b** demonstrate the following contours as delineated on MR fusion (**a**) and planning CT (**b**): GTV – blue; High Risk PTV (HRPTV) – red; Bladder - pink; Intermediate Risk PTV (IRPTV) – purple and Low Risk PTV (LRPTV) – light brown. Figs. **c** and **d** show these contours overlaid on the cone beam CT to facilitate soft tissue match

For patients planned with an empty bladder a hand-held ultrasound was performed daily prior to treatment to ensure the bladder was empty. A CBCT was obtained prior to treatment with the Varian Trilogy on board imaging system with a 2.5 mm slice thickness over a 16 cm length with a resolution of 384 × 384 pixels. Following this the treating radiation therapists performed a soft tissue match to the GTV. This was supervised by the treating Radiation Oncologist for the first five fractions, and thereafter as required based upon feedback from the radiation therapist. The first step was to identify if the bladder was outside the IRPTV, in which case these patients were asked to further empty their bladder. If present, fiducial markers were used to aid initial alignment in the superior/inferior direction, and then the GTV was matched. If no fiducials were present then the GTV was used to guide the entire match (see Fig. 1). This match was centred on the region of the bladder identified as the highest risk of gross disease (GTV), rather than identifying any abnormality on the CBCT itself. In patients treated with a full bladder a margin of 10 mm above and below the superior aspect of the bladder was defined on the planning scans. Patients were required to have a bladder volume within these limits on CBCT before proceeding with the match to the GTV.

### Assessment and follow up

Patients were assessed weekly during treatment and 3-6 monthly after treatment. Toxicity was graded by the treating physician at each assessment according to the Common Terminology Criteria for Adverse Events (CTCAE) version 4.0. Cystoscopy was performed 6-12 weeks post treatment and annually thereafter. CR was defined as no visible evidence of bladder tumour and a negative biopsy. The primary endpoint for the study was freedom from local failure (FFLR). Secondary endpoints were complete response rate on cystoscopy, acute and late toxicity and overall survival.

### Statistics

Survival outcomes were calculated from the first day of radiation treatment to the first clinical, radiologic or cystoscopic evidence of recurrence in the bladder (FFLR), any recurrence (disease free survival, DFS) or death (OS). In the absence of any of these events, survival was censored at the last follow up. The rates of FFLR, DFS and OS and were calculated using the Kaplan-Meier method.

## Results

### Patient characteristics

Patient and tumour characteristics are outlined in Table 1. In total, twenty-eight patients were treated on this protocol between June 2010 and September 2013. The cohort included 22 males and six females, with a median age of 83 years (range 58-92). All but two patients had muscle-invasive transitional cell carcinoma; two others had squamous cell carcinoma. Stage T2, T3 and T4 disease was identified in 6, 14 and 8 patients respectively. Five patients had positive nodes, and four patients had distant metastatic disease. A maximal TURBT was possible in 64 % of cases and 32 % had hydronephrosis at presentation. A further 36 % had a history of previous superficial bladder cancer receiving intravesical BCG.

**Table 1:** Patient and tumour characteristics

Characteristic	n (%)
Gender	
Female	6 (21)
Male	22 (79)
Age (y)	
Median	83
Range	58-92
ECOG	
0	14 (50)
1	10 (36)
2	4 (14)
Histology	
TCC	26 (93)
SCC	2 (7)
T stage	
T2	8 (29)
T3	16 (57)
T4	4 (14)
Overall stage	
II	6 (21)
III	14 (50)
IV	8 (29)
TURBT	
Complete	18 (64)
Incomplete	10 (36)
Hydronephosis	
Yes	9 (32)
No	19 (68)
Previous BCG	
Yes	9 (32)
No	19 (68)

### Treatment and toxicity

Radiation and chemotherapy treatment details are outlined in Table 2. In 23 patients, radiation treatment was given with curative intent with ten patients receiving concurrent chemotherapy (cisplatin n = 8; carboplatin n = 2) and 13 patients receiving radiation alone. In five patients, who had either extensive nodal involvement or metastatic disease at diagnosis, radiation treatment was considered palliative. These patients received a dose of 50-55 Gy in 20 fractions. A total of 22 patients received treatment to the regional lymph nodes, including all patients having concurrent chemotherapy. The GTV was identified and treated with an integrated boost in all but one patient. Fiducial markers were successfully placed in nine patients. There were a total of 680 fractions delivered to the 28 patients. Seventeen patients were treated with an empty bladder and eleven with a full bladder. Average treatment time, including set-up was 11 min, with patients scheduled in standard 15-min bookings.

**Table 2:** Treatment details

	Treatment group (n)			
	All	CRT	Definitive RT	Palliative RT
	(n = 28)	(n = 10)	(n = 13)	(n = 5)
20 fractions	17	0	12	5
30 fractions	11	10	1	0
GTV identified	27	10	12	5
LNs treated	22	10	9	3

All patients completed radiation treatment as prescribed. Reproducibility of bladder filling on the CBCT was very good with an empty bladder, with only five of 400 fractions requiring patients to be removed from the treatment couch. The full bladder protocol was less reproducible, with seven of the 11 patients requiring at least one re-set up to fill their bladders. In total, re-set up was required for 29 of 280 fractions in the full bladder group, 22 due to inadequate filling, five due to overfilling, one due to machine breakdown and one due to gas obscuring the GTV match. Of the 11 patients who commenced treatment with a full bladder plan, two were subsequently replanned with empty bladders, one at fraction 5 and one at fraction 10.

Three patients ceased concurrent chemotherapy early, two due to cisplatin-related emesis, and another due to Grade 2 thrombocytopenia. Four patients required admission to hospital during treatment. Two of these admissions were for supportive care and intravenous fluids due to treatment toxicity. A further two patients were admitted overnight for packed-cell transfusion to treat symptomatic anaemia resulting from haematuria which had been present at baseline. In both cases, the admissions took place in the first half of radiation treatment and the haematuria subsequently resolved.

Acute Grade 2 GU toxicity was reported by six patients (21 %). No patients experienced acute Grade 3 GU toxicity. Six patients reported Grade ≥2 GI toxicity, including two patients with grade 3 nausea. There were five patients (18 %) with Grade ≥2 late GU toxicity including one patient requiring bilateral ureteric stents (Grade 3). There was no late Grade 2 or 3 GI toxicity. These figures are summarised in Table 3.

**Table 3:** Genitourinary (GU) and Gastrointestinal (GI) treatment toxicity

	Worst CTCAE grade, any event (n)			
	0	1	2	3
Acute toxicity				
- GU	4	18	6	0
- GI	8	14	4	2
Late toxicity				
- GU	10	13	4	1
- GI	17	11	0	0

*n* number of patients, *CTCAE* Common Terminology Criteria for Adverse Events

### Outcomes

Patients were analysed according to the intent of treatment (definitive versus palliative). Within the definitive group, subset analyses were performed for patients treated with chemoradiation and those who received radiation alone. Results are summarised in Table 4.

**Table 4:** Outcomes according to treatment group

	Treatment group n(%)			
	Total	CRT	Definitive RT	Palliative
Cystoscopy	22	9	10	3
CR^a^	19 (86)	9 (100)	8 (80)	2 (60)
Failure	8	0	5	3
- Local	4	1	2	1
- Distant	3	0	1	2
- Local and distant	1	0	1	0
Death	8	0	5	3
- From bladder cancer	5	0	3	2
- From other cause	3	0	2	1

^a^CR = % of patients who underwent cystoscopy and had a complete response

### Definitive cohort

Nineteen of the 23 definitive patients underwent cystoscopy and biopsy following treatment, including nine of the ten patients who received CRT and ten of 13 patients in the RT alone group. Of the four patients who did not have cystoscopy, one was lost to follow up overseas and three patients treated with RT alone declined the procedure. The cystoscopic complete response rate was 86 %, (100 % in the CRT group and 81 % in the radiotherapy alone group). At 2 years the local control was 90 % in the CRT group and 86 % in the definitive RT alone group and OS was 100 % and 69 % respectively.

**Fig. 2:**
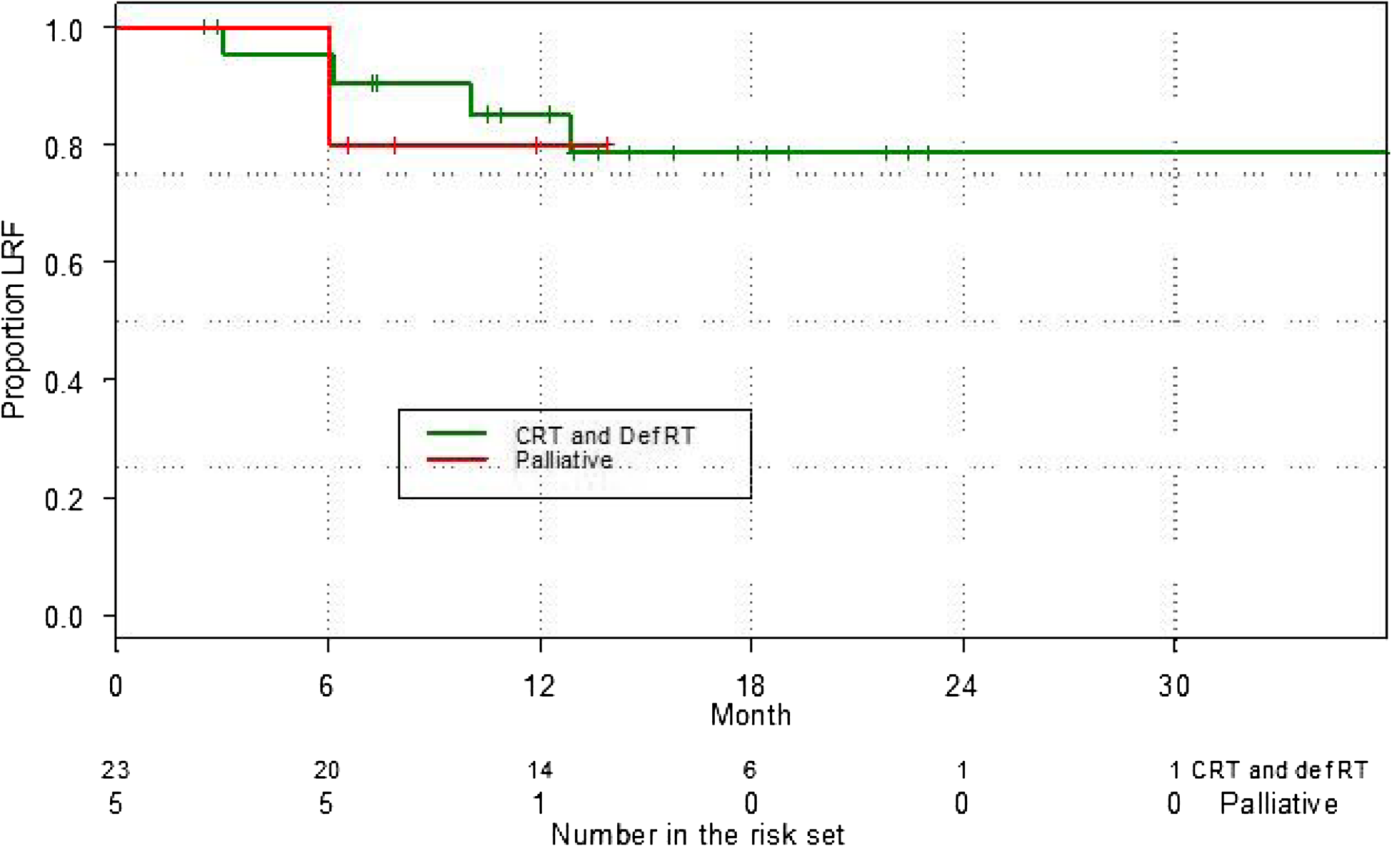
Kaplan Meier curve of Freedom from Locoregional Recurrence (FFLR) for definitive and palliative patients

**Fig. 3:**
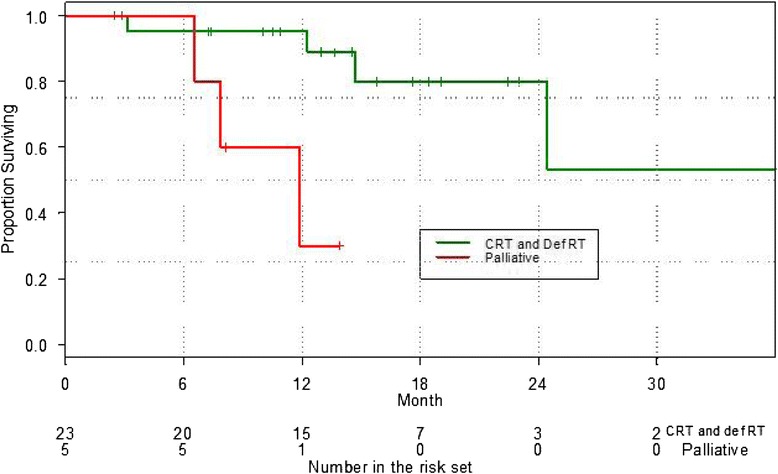
Overall survival for definitive and palliative patients

In patients treated definitively, freedom from local failure was 78 % at 2 years (Fig. 2). There were four local failures, with one patient proceeding to salvage cystectomy. Of the remaining three patients, two declined intervention and remain alive with disease; one patient has died as a result of obstructive renal failure. A further two patients in the group have had documented distant relapse. The DFS and overall survival rates were 73 % and 80 % at 2 years respectively. Kaplan Meier curves for overall survival are shown in Fig. 3.

### Palliative cohort

Three of the five patients treated with palliative intent died during the follow up period, two due to bladder cancer and one from an unrelated illness. One patient experienced local progression, translating to a 1 year FFLR of 80 %, and a median of 6 months. Median survival was 10 months (1 year OS 30 %).

## Discussion

In our series, IG-IMRT with a synchronous integrated boost was well tolerated, with excellent early results. Although longer follow up is required, the high complete response rate and 2 year loco-regional control support the feasibility and promise of this technique.

The heterogeneous nature of our cohort in terms of tumour stage and treatment is acknowledged. However, allowing for this, our results compare favourably to larger bladder preservation studies employing 3D conformal techniques and minimal image guidance. In the landmark BC2001 study of chemoradiation with mitomycin and 5-fluorouracil versus radiation alone, James et al. reported a 2-year locoregional DFS of 67 % in the chemoradiotherapy group, and 54 % in the radiotherapy alone group [5]. In two sequential phase II trials of definitive chemoradiation with weekly low dose cisplatin conducted by the Trans Tasman Radiation Oncology Group (TROG) Gogna et al. reported a CR rate of 70 % and local control of 45 % at 5 years [22]. This compares to our cohort who had a 90 % 2 year local control following chemoradiotherapy and 86 % for radiotherapy alone.

Although there is limited literature on the use of IMRT for bladder cancer, two studies that are available report similar outcomes. Turgeon et al. reported a CR rate of 83 % and a 2 year OS of 69 % in a series of 24 patients treated with hypofractionated IMRT and concurrent chemotherapy [23]. In a separate prospective study Hsieh et al. described a 2-year locoregional recurrence free survival of 87.5 % in 19 patients with bladder cancer who were treated with either IMRT or tomotherapy. The overall survival in their cohort was low (26.3 % at 2 years) reflecting the fact that one third of the cohort had stage IV disease [24]. Of note neither of these studies used daily soft tissue matching, and PTV margins were generous at 1.5 to 2.5 cm. The addition of daily soft tissue matching in our protocol allowed a margin reduction to 0.5 cm for high-risk areas and 1 cm around the bladder. With the use of IMRT and small margins, relative dose escalation was possible while achieving low toxicity (see Table 3).

Palliative cases notwithstanding, our cohort as a whole represents a high-risk population. The majority of the cases were stage III/IV, and overall the patients were elderly (median age 83 years). One third of patients had hydronephrosis, incomplete TURBT or a history of superficial carcinoma progressing despite BCG, all of which are known poor prognostic indicators [25–28]. In this context, our 2 year FFLR and OS for the definitive cohort (78 % and 80 % respectively) are very encouraging. Therefore although we have demonstrated excellent outcomes for a highly selected group of patients who received combined chemoradiation, high dose radiation need not be reserved for these patients alone.

The acute toxicity observed in our cohort was modest compared to rates reported in other high dose trials. James et al. reported CTCAE grade 3-4 acute side effects in 27.5 % and 36 % in the radiotherapy alone and chemoradiotherapy groups respectively [5]. In the TROG chemoradiation trials, 35 % of patients experienced grade 2 GI effects, and more than 40 % reported grade 2 GU toxicity [22]. These trials used conformal radiation techniques, and we note that the low GI toxicity experienced by our patients is in keeping with the normal tissue sparing properties of pelvic IMRT [23, 24]. More specifically, clinical and planning studies have demonstrated the superiority of IMRT compared with conformal therapy with respect to small bowel volumes receiving doses of 20-50Gy [29, 30]. This is of particular relevance to patients in whom the clinical target extends beyond the bladder to include the regional nodes, as was the case in the majority of our patients. Although pelvic nodal relapse following bladder radiotherapy is reportedly uncommon, the large margins employed for the radiation delivered in previous trials may have resulted in the majority of the regional nodes receiving therapeutic radiation doses. In our cohort, nodal irradiation was justified on the basis of the comparatively small margins (1 cm) used in our technique and evidence from surgical series demonstrating improved outcome with nodal dissection [31].

Our patients received a simultaneous integrated boost to the GTV, identified using clinical, cystoscopic and radiologic information. We found multiparametric MRI to be a substantial aid in GTV delineation. The main limitation with MRI fusion was the requirement for a comfortably full bladder at the time of MRI to aid diagnostic reporting, with most patients having their final volumes on an empty bladder scan. We are currently investigating elastic deformation to account for the anisotropic margins required for changes in bladder size, which will enable better fusion of the MRI and planning CT. MRI is not only promising with respect to bladder tumour staging and characterisation, but also as a potential predictor of pathologic response to therapy [32, 33].

An alternative means to identify the GTV is fiducial markers, which were successfully placed around the tumour bed in nine of our patients. Elsewhere, the use of fiducial markers has been reported with few complications and excellent visibility on CBCT [34–36]. However, in our experience, the implementation of a fiducial marker program for bladder radiation proved to be a challenging process and did not always aid in tumour delineation. As such it was ceased part way through the study. Custom-designed equipment as described by Garcia et al. may address some of the technical difficulties that inhibited marker placement in many of our patients [34].

Various adaptive strategies have been described by several groups including ‘PTV of the day’ selection, isocentre shifts and corrections for translational and rotational motion [12, 14, 37, 38]. Our daily image-guidance with CBCT and soft tissue match is unique with no previously published IMRT series utilising this approach. It is clear from the extensive experience in other pelvic malignancies that as the quality of on-board CBCT imaging has improved, so has the workflow and ease of matching. In our patients, on average treatment was completed well within the allocated time, and therefore did not impact adversely on departmental resources or workflow. The treating Radiation Oncologist supervised the matching during the first week of treatment and only attended subsequent to this if problems were identified by the radiation therapy team, including the seven occasions when reset up was required and two patients who ultimately needed replanning.

In a paper comparing matching techniques in patients treated on an adaptive planning bladder study, Foroudi et al. found 1 cm CTV to PTV margins were found to be feasible when using soft tissue match. The resulting coverage with this margin was adequate in 89 % of cases, whereas margins of 2 cm or greater were required to achieve the same coverage using bony alignment or skin tattoos [39]. Our study further supports this finding, with only 5/400 fractions treated with an empty bladder requiring the patient to be taken off the table. We believe this high rate of daily bladder filling reproducibility is a result of patient education with respect to bladder emptying and subsequent reinforcement by daily feedback from the treating therapists based on ultrasound and CBCT images. This is in contrast to the large variations in bladder filling reported from the recent TROG study: 16 % of patients in that study had, on at least one occasion, a pre-treatment bladder CTV extending beyond all of the three possible adaptive plans [37].

A limitation of our study was no CBCTs were performed after treatment to assess intrafraction motion which may be important, especially with the high dose gradients of IMRT which could be more pronounced on days of hydration for cisplatin chemotherapy [37]. The risk is the bladder may move outside the IRPTV for part of the beam-on time. Our excellent local control, despite this risk, supports the concept that not all regions of the bladder require the same dose. This was shown in the BC2001 study. In this study there was similar 2-year local control with partial bladder irradiation (64 %) and full bladder irradiation (61 %) [40]. Intrafraction motion will also be reduced in the future with the introduction of volumetric arc therapy (VMAT) [41].

## Conclusion

IG-IMRT with daily soft tissue matching is a promising approach in the treatment of muscle invasive bladder cancer, enabling the delivery of an accelerated simultaneous integrated boost IMRT with low rates of toxicity. Early local control and disease free survival are encouraging, especially in light of our high-risk population. Longer follow-up is required to assess the efficacy of this technique.

## Abbreviations

CBCT: Cone beam CT
CR: Complete response
CRT: Chemoradiation
CTV: Clinical target volume
DFS: Disease free survival
FFLR: Freedom from locoregional recurrence
GI: Gastrointestinal
GTV: Gross tumour volume
GU: Genitourinary
HRPTV: High risk planning target volume
IG-IMRT: Image-guided intensity modulated radiotherapy
IRCTV: Intermediate risk clinical target volume
IRPTV: Intermediate risk planning target volume
LRPTV: Low risk planning target volume
SIB: Simultaneous integrated boost
TURBT: Transurethral resection of bladder tumour
